# Bio-Inspired Photocatalytic Nitrogen Fixation: From Nitrogenase Mimicry to Advanced Artificial Systems

**DOI:** 10.3390/nano15191485

**Published:** 2025-09-29

**Authors:** Wenpin Xia, Kaiyang Zhang, Jiewen Hou, Huaiyu Fu, Mingming Gao, Hui-Zi Huang, Liwei Chen, Suqin Han, Yen Leng Pak, Hongyu Mou, Xing Gao, Zhenbin Guo

**Affiliations:** 1 College of Biological and Chemical Engineering, Qilu Institute of Technology, Jinan 250200, China; 2 Advanced Technology Research Institute, Beijing Institute of Technology, Jinan 250300, China

**Keywords:** photocatalytic nitrogen fixation, bio-inspired catalysis, nitrogenase mimicry, ammonia synthesis, defect and interface engineering, solar-driven artificial nitrogenase

## Abstract

Photocatalytic nitrogen fixation under ambient conditions offers a sustainable alternative to the energy-intensive Haber–Bosch process, yet remains limited by the inertness of N≡N bonds and sluggish multi-electron/proton transfer kinetics. Nature’s nitrogenase enzymes, featuring the FeMo cofactor and ATP-driven electron cascades, inspire a new generation of artificial systems capable of mimicking their catalytic precision and selectivity. This review systematically summarizes recent advances in bio-inspired photocatalytic nitrogen reduction, focusing on six key strategies derived from enzymatic mechanisms: Fe–Mo–S active site reconstruction, hierarchical electron relay pathways, ATP-mimicking energy modules, defect-induced microenvironments, interfacial charge modulation, and spatial confinement engineering. While notable progress has been made in enhancing activity and selectivity, challenges remain in dynamic regulation, mechanistic elucidation, and system-level integration. Future efforts should prioritize operando characterization, adaptive interface design, and device-compatible catalyst platforms. By abstracting nature’s catalytic logic into synthetic architectures, biomimetic photocatalysis holds great promise for scalable, green ammonia production aligned with global decarbonization goals.

## 1. Introduction: Green Ammonia and the Biomimetic Revolution

Ammonia (NH_3_) is a cornerstone of the global chemical industry, primarily utilized in fertilizers, but also increasingly seen as a potential hydrogen carrier and carbon-free energy vector in the transition toward net-zero emissions [1,2,3]. However, the conventional Haber–Bosch process, responsible for more than 150 million tons of annual NH_3_ production, consumes ~2% of the world’s total energy supply and emits over 1.5% of global CO_2_ emissions due to its dependence on fossil fuels and high-temperature/high-pressure conditions [4,5,6]. This contradiction between ammonia’s industrial importance and its unsustainable synthesis route has catalyzed intensive exploration of alternative green production methods [7].

Among the various routes proposed, photocatalytic nitrogen reduction reaction (NRR) under ambient conditions has emerged as one of the most attractive solutions, enabling solar-driven conversion of atmospheric N_2_ into NH_3_ without external hydrogen or harsh conditions [8,9]. Despite growing interest, most artificial photocatalysts still suffer from a low NH_3_ yield, which stem from the inertness of the N≡N triple bond (941 kJ mol^−1^) and the complex multi-electron/proton transfer process [10,11].

Nature, however, offers an elegant and efficient solution: nitrogenase enzymes, particularly the molybdenum-dependent nitrogenase featuring the FeMo cofactor (FeMoco) [12,13,14]. These enzymes catalyze N_2_ reduction at ambient conditions with high selectivity and turnover frequency, facilitated by a cascade of multi-metallic clusters (P-cluster, M-cluster) and ATP-driven electron/proton transfer pathways [15,16,17,18,19,20]. Nitrogenase-based systems serve as blueprints for a new generation of artificial catalysts, inspiring the development of bio-inspired photocatalysts that mimic the structural, electronic, and functional features of the natural enzyme [21,22,23].

In recent years, a surge of research has explored strategies to emulate nitrogenase catalysis using engineered nanomaterials. These include constructing metal–sulfur clusters analogous to FeMoco [24,25], designing electron relay pathways akin to P→M clusters [26,27], and incorporating defect structures to mimic flexible reaction pockets [28,29]. More advanced systems even integrate quantum dots or molecular photosensitizers to mimic the ATP-driven energy supply in nitrogenase catalysis [30]. Collectively, these systems incorporate “bio-logic” features such as spatially separated charge centers, multi-site synergy, and adaptive proton/electron relay—pushing photocatalytic NRR systems toward biomimetic performance [31,32].

This review aims to provide a comprehensive and systematic overview of the current advances in bio-inspired photocatalytic nitrogen fixation, with a particular focus on the design strategies, material innovations, and mechanistic insights that draw from nitrogenase chemistry. We first outline the biological foundation of N_2_ fixation, detailing the structural and functional principles of natural nitrogenases. Next, we classify and discuss key design strategies in biomimetic photocatalyst development, including FeMo active-site simulation, electron pathway engineering, artificial ATP-mimicking systems, defect regulation, and multi-site synergism. Finally, we summarize critical challenges and offer perspectives for future research that may enable scalable, sustainable, and enzyme-level efficient photocatalytic ammonia production.

## 2. Industrial and Biological Context of Ammonia Synthesis

### 2.1. Societal and Industrial Applications of Ammonia

Ammonia is one of the most important chemicals in modern society, with annual global production exceeding 180 million metric tons. Its dominant use lies in fertilizers (urea, ammonium nitrate, and ammonium sulfate), which sustain food production for more than 40% of the world’s population. Beyond agriculture, ammonia serves as a critical feedstock for diverse chemicals, including plastics, explosives, and pharmaceuticals.

In recent years, ammonia has also attracted growing attention as a carbon-free energy vector. With a high hydrogen content (17.6 wt%) and established global infrastructure for storage and transport, ammonia can act as a hydrogen carrier or be directly used in solid oxide fuel cells and combustion engines. Pilot-scale projects are already investigating ammonia co-firing in power plants and its role in green hydrogen economies.

These multifaceted applications reinforce the importance of developing sustainable N_2_ fixation technologies. Photocatalytic ammonia synthesis, if realized at scale, could provide not only decentralized fertilizer production but also a renewable pathway toward future energy systems.

### 2.2. From Haber–Bosch Process to Nitrogenase Catalysis

The Haber–Bosch process, established in the early 20th century, remains the cornerstone of industrial ammonia production. The overall reaction follows:

N_2_(g) + 3H_2_(g) ⇌ 2NH_3_(g), ΔH = −92.4 kJ mol^−1^

Despite its apparent simplicity, this reaction is thermodynamically and kinetically challenging. Activation of the N≡N triple bond requires high temperature (400–500 °C) and high pressure (150–300 bar), typically over Fe-based catalysts promoted with K_2_O, CaO, or Al_2_O_3_. The H_2_ feedstock is primarily derived from steam reforming of methane (CH_4_ + H_2_O → CO + 3H_2_), which contributes substantially to CO_2_ emissions. As a result, Haber–Bosch consumes nearly 1–2% of global energy supply and contributes more than 300 Mt CO_2_ annually.

**Figure 1:**
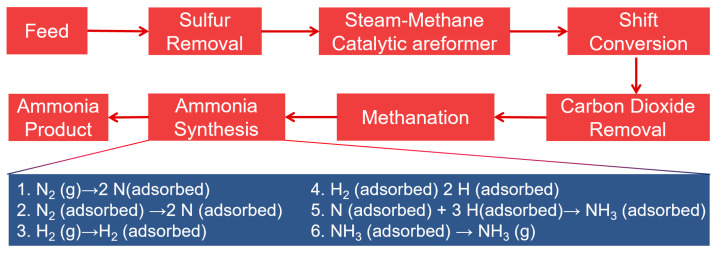
Simplified schematic of the Haber–Bosch process.

A schematic of the process (Figure 1) illustrates the main stages: (i) CH_4_ reforming and H_2_ generation, (ii) N_2_ separation from air, (iii) high-temperature and high-pressure catalytic synthesis, and (iv) continuous recycling of unreacted gases. While this process sustains global agriculture, its extreme energy demands and environmental footprint highlight the urgent need for sustainable alternatives such as biomimetic photocatalytic N_2_ fixation.

The industrial Haber–Bosch process remains unmatched in terms of ammonia yield, with global plants typically producing 1500–2000 tons NH_3_ per day at an energy cost of 8–12 MWh per ton NH_3_, depending on feedstock and plant efficiency (Table 1). The reliance on methane-derived hydrogen also makes it carbon intensive, emitting 1.6–2.0 tons CO_2_ per ton NH_3_ produced. While the cost per ton is relatively low (≈300–600 USD), the environmental externalities are significant.

In contrast, current bio-inspired and photocatalytic nitrogen fixation systems achieve yields in the order of μmol–mmol NH_3_ g^−1^ h^−1^, far from industrial scales. However, they operate under ambient temperature and pressure without fossil-derived hydrogen input, offering the potential for decentralized, renewable-powered ammonia synthesis with drastically reduced marginal costs once efficient catalysts and device integration are realized. Recent techno-economic analyses suggest that if photocatalytic systems achieve ≥10 mmol g^−1^ h^−1^ rates with solar-to-ammonia efficiencies of 1–2%, the levelized cost of ammonia could become competitive with Haber–Bosch, particularly in regions with abundant solar resources.

## 3. Natural Nitrogenase Systems: Structural and Mechanistic Inspirations

Biological nitrogen fixation is accomplished by nitrogenase enzymes—nature’s only known catalysts capable of converting inert N_2_ into NH_3_ under ambient conditions. Among the three known nitrogenase isoforms (Mo, V, and Fe-only), the Mo-dependent nitrogenase is the most well-studied and catalytically efficient [15,16]. It consists of two key protein components: the dinitrogenase reductase (Fe protein) and the dinitrogenase (MoFe protein). The Fe protein mediates ATP-dependent electron delivery, while the MoFe protein houses the active site FeMo cofactor (FeMoco), a complex [MoFe_7_S_9_C] cluster responsible for N_2_ activation and reduction [17,18].

### 3.1. Structural Insights: FeMoco and P-Cluster Architectures

The FeMoco cofactor is composed of a Mo atom coordinated by three sulfur atoms and a homocitrate ligand, linked to a [Fe_7_S_9_C] cluster containing a central interstitial carbon atom. This unique cofactor is embedded within the MoFe protein, forming a precisely oriented reaction pocket that allows for N_2_ binding, H^+^ delivery, and e^−^ transfer in a concerted manner [19]. Prior to FeMoco, the electron relay occurs through the P-cluster, an [Fe_8_S_7_] double-cubane structure that bridges the Fe protein and the FeMoco site [20]. The P-cluster undergoes conformational and redox changes during the electron transfer process, functioning as a molecular switch or gate [33].

High-resolution crystallographic studies (1.0–1.3 Å) have revealed the spatial arrangement and flexibility of these clusters, shedding light on how enzyme-controlled confinement and geometry affect catalysis [18,34]. Such hierarchical structures have motivated efforts to emulate multi-metallic clusters, coordinated ligand environments, and flexible cavity-based activation sites in artificial systems.

### 3.2. Mechanistic Models: From Lowe–Thorneley to New Paradigms

**Figure 2:**
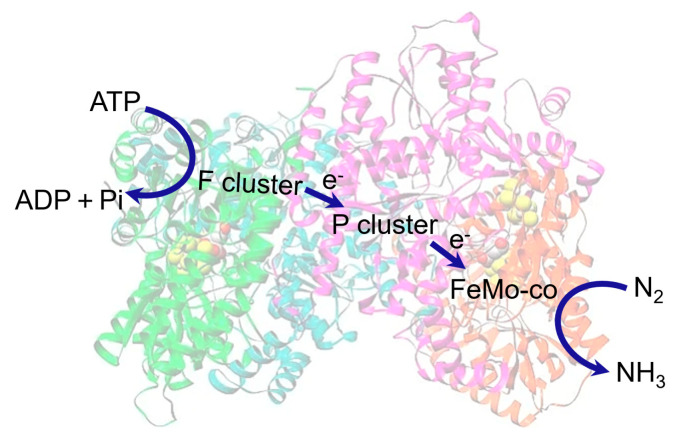
Schematic illustration of N_2_ reduction and electron transfer within the nitrogenase complex.

The classic Lowe–Thorneley (LT) model describes nitrogenase catalysis as a stepwise, proton-coupled electron transfer (PCET) process, in which the FeMo-cofactor (FeMoco) cycles through a series of reduced states (*E*_0_–*E*_8_) to accumulate the 8e^−^/8H^+^ equivalents required for N_2_ fixation [21]. During the early stages (*E*_0_–*E*_4_), electrons and protons delivered from the Fe protein via the P-cluster ([8Fe–7S]) are stored as bridging hydrides within FeMoco. Once sufficient reducing equivalents are accumulated, N_2_ binding occurs, most likely at an Fe6 site adjacent to the interstitial carbide (Figure 2).

The activation of N_2_ is exceptionally challenging due to the high bond dissociation energy of the N≡N triple bond (941 kJ mol^−1^) and the need for precise temporal control of multi-electron/proton delivery. Stepwise PCET weakens the N≡N bond through formation of key intermediates (e.g., N_2_H, N_2_H_2_, N_2_H_4_), eventually yielding two NH_3_ molecules. This process is frequently accompanied by obligatory H_2_ evolution, reflecting the delicate balance between desired N_2_ reduction and competing side reactions [35].

Recent experimental and computational insights have expanded the LT framework, suggesting alternative pathways involving early N_2_ binding, side-on coordination geometries, and the critical role of the interstitial carbide in tuning Fe electronic states [22]. Together, these advances emphasize not only the unique structural features of FeMoco but also the pivotal electron-relay function of the P-cluster, offering mechanistic lessons for the rational design of biomimetic and artificial NRR catalysts.

When benchmarked against the two natural and industrial references, artificial photocatalytic systems remain at an early stage. Nitrogenase catalyzes N_2_ reduction under ambient conditions with remarkable selectivity but requires ATP hydrolysis, limiting its turnover frequency. In contrast, Haber–Bosch achieves industrially relevant yields but at the cost of extreme temperature–pressure input and substantial carbon emissions. Artificial systems currently achieve μmol-scale production rates and modest quantum efficiencies under ambient conditions, highlighting both their unique promise and critical gap. Thus, bridging the enzymatic selectivity of nitrogenase with the scalability of Haber–Bosch remains the central challenge for next-generation catalysts (Table 2).

### 3.3. Role of ATP and Electron Delivery Pathways

A hallmark feature of biological nitrogen fixation is the ATP-coupled electron transfer from the Fe protein to the MoFe protein. Each electron transfer requires the hydrolysis of two ATP molecules, serving not only as an energy source but also to trigger structural rearrangement and docking of the protein units [16,17]. This energy input facilitates efficient e^−^ injection into the P-cluster and subsequently into the FeMoco site [19].

In artificial systems, mimicking this complex ATP-driven cascade remains a grand challenge. Nonetheless, the concept of spatially controlled electron flow and gated transfer between cofactor analogs has led to the use of photosensitizers, semiconductor–metal junctions, and redox mediators in bio-inspired catalyst design.

### 3.4. Design Implications for Artificial Systems

The architecture and function of nitrogenase enzymes provide several pivotal insights for designing biomimetic photocatalysts: (i) active center mimicry: replicating the geometry and electronic structure of FeMoco using multi-metallic clusters (e.g., Fe–Mo–S motifs); (ii) redox relay networks: constructing P-cluster-like units or conductive linkers for sequential electron transfer; (iii) cavity and ligand effects: engineering confined or defect-rich sites that facilitate N_2_ adsorption and PCET; (iv) energy coupling strategies: integrating ATP-analog pathways using light, voltage, or redox molecules [36,37,38,39]. These design elements are increasingly reflected in advanced catalyst architectures discussed in Section 3 and Section 4 [40,41].

## 4. Bio-Inspired Catalyst Design Strategies for Photocatalytic NRR

### 4.1. Biomimetic Construction of FeMo-like Active Centers

In biological nitrogen fixation, the FeMo cofactor (FeMoco), a highly complex [MoFe_7_S_9_C] cluster, serves as the catalytic epicenter of nitrogenase enzymes [17,18]. It integrates heteronuclear Fe–Mo active sites, sulfur-bridged coordination, and confined electronic environments, enabling stepwise proton-coupled multi-electron N≡N bond cleavage under ambient conditions. Mimicking such a multifunctional unit in artificial photocatalysts is a grand challenge, yet crucial for designing enzyme-like activity and selectivity [23,24,42].

#### 4.1.1. Bionic Fe–Mo Architectures on Layered Semiconductors

**Figure 3:**
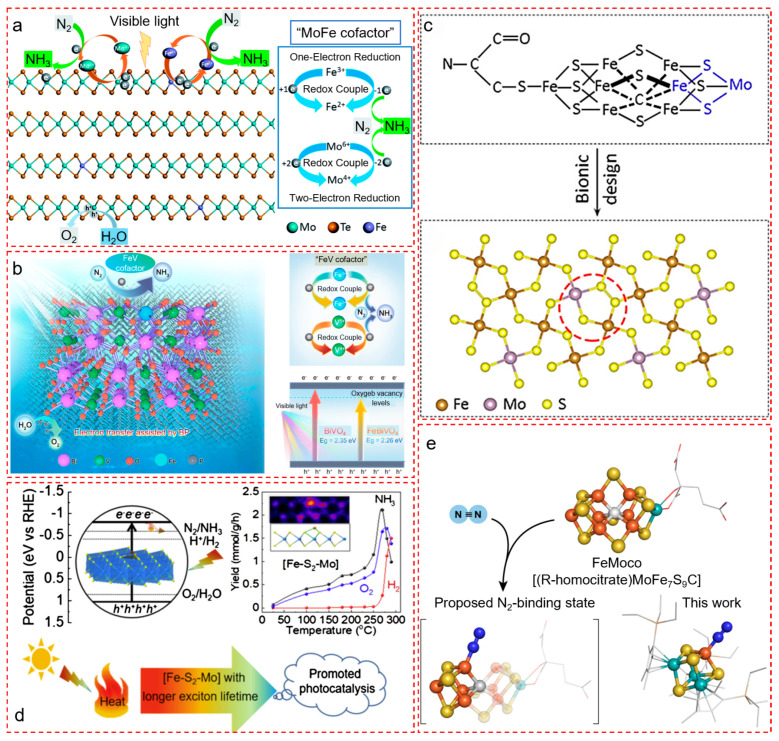
Representative strategies for active-site mimicry in photocatalytic nitrogen fixation: structural and electronic emulation of the FeMo cofactor. Reproduced with permission. (**a**) Proposed photocatalytic N_2_ reduction pathway over a Fe–MoTe_2_ heterostructure under visible light. The Fe–Mo dual-site interface replicates the binuclear arrangement of FeMoco, enabling synergistic adsorption and activation of N_2_ via charge redistribution. Reproduced with permission [43]. Copyright © 2020 Royal Society of Chemistry. (**b**) Schematic representation of the FeBiVO_4_-0.05BP photocatalyst. The Fe active site simulates the iron center of nitrogenase, while BiVO_4_ provides a visible-light-absorbing matrix. The dual-functional architecture facilitates stepwise N_2_ protonation and suppresses competing HER via proton channel redirection. Adapted from ref. Reproduced with permission [44]. Copyright © 2023 Royal Society of Chemistry. (**c**) Molecular and structural design of Mo(IV)-doped FeS_2_. Upper panel: structural analogy to the FeMo cofactor of nitrogenase with [Fe–S_2_–Mo] motifs; lower panel: schematic of FeS_2_ with Mo substitution, which induces Fe–Mo–S coordination resembling FeMoco subunits and enhances N_2_ adsorption capacity. Reproduced with permission [45]. Copyright © 2020 American Chemical Society. (**d**) Atomic-scale imaging and model of Fe single-atom anchored on sMoS_2_. The Fe_1_ center occupies a Mo atop site in a trigonal prismatic environment, forming a [Fe–S_2_–Mo] unit analogous to enzymatic cofactor geometry. The system exhibits strong photothermal synergy in NRR, with photogenerated excitons extended by thermally activated carrier lifetimes. Reproduced with permission [46]. Copyright © 2021 Cell Press. (**e**) Schematic of K^+^-intercalated crystalline carbon nitride (KCCN) containing nitrogen vacancies (NVs) for visible-light-driven nitrogen photofixation. NVs chemisorb and activate N_2_, while intercalated K^+^ enables robust electron storage within the carbon nitride framework [47]. Copyright © 2022 Elsevier.

To mimic the MoFe synergistic centers in FeMoco, researchers have constructed heteroatomic Fe/Mo frameworks embedded in low-dimensional semiconductors. Li et al. designed a bio-inspired catalyst by doping Fe atoms into two-dimensional MoTe_2_ nanosheets, forming an in-built “MoFe-cofactor” that emulates the redox-active nature of FeMoco (Figure 3a) [43]. The Fe dopants modulate the MoTe_2_ electronic structure via mixed-valence redox couples (Fe^3+^/Fe^2+^ and Mo^6+^/Mo^4+^), enhancing photogenerated electron–hole separation and prolonging the carrier lifetime by 15-fold. This significantly boosts photocatalytic N_2_ reduction activity—exhibiting an 11-fold enhancement over pristine MoTe_2_. The system represents a functional MoFe analog in a 2D inorganic matrix, retaining photo-electrochemical synergy through lattice-level redox engineering [25,42].

In a similar approach, Li et al. reported a bionic “FeV-cofactor” catalyst by doping Fe into BiVO_4_ and decorating it with few-layer black phosphorus (BP) nanosheets (Figure 3b) [44]. The Fe centers act as biomimetic N_2_ activation sites, while BP enhances interfacial charge transfer and broadens visible-light absorption. Density functional theory (DFT) analysis revealed that Fe doping facilitates N_2_ adsorption and activation, while V^4+^/V^5+^ and Fe^2+^/Fe^3+^ couples promote charge redistribution. The resulting FeBiVO_4_–0.05BP achieved a high NH_3_ yield of 337.9 μmol g^−1^ h^−1^, ranking among the best oxide-based photocatalysts. This study underlines the importance of cofactor-inspired multi-metal synergy and redox-coupled band modulation.

#### 4.1.2. Atomically Precise Fe–Mo–S Motifs on Sulfide Matrices

To mimic the precise Fe–S–Mo bonding geometry in FeMoco, researchers have introduced Fe or Mo atoms into sulfur-rich matrices with atomic-level control. Wang et al. synthesized Mo(IV)-doped FeS_2_ nanocrystals, where Mo atoms replace Fe sites to form [Mo–S–Fe] analogs of the enzymatic cofactor (Figure 3c) [45]. The Mo dopants not only suppress competing hydrogen evolution but also enrich the active site density and promote N_2_ adsorption. The resulting electrocatalyst achieved a Faradaic efficiency of 14.41% at −0.2 V vs. RHE, highlighting the effectiveness of Mo-centered electron delocalization within an Fe–S lattice. This reflects the essential role of heteronuclear metal synergy and sulfur coordination in facilitating N≡N bond weakening.

In another approach, Zheng et al. engineered a catalyst where Fe atoms are anchored onto monolayer MoS_2_, creating [Fe–S_2_–Mo] motifs on a molecularly thin support (Figure 3d) [46]. This configuration emulates the structural pattern of FeMoco, with Fe atoms occupying Mo sites and forming trigonal prismatic arrangements with sulfur. Photocatalytic N_2_ reduction under visible light was significantly enhanced at elevated temperatures, reaching 0.24% solar-to-ammonia conversion efficiency—the highest reported among inorganic systems. The results demonstrate that spatially confined metal–sulfur coordination environments can replicate enzymatic charge localization and orbital overlap essential for N_2_ activation [27,46,47,48].

#### 4.1.3. Synthetic Cubic Fe–Mo–S Clusters Emulating FeMoco Geometry

Going beyond surface modification, Ohki et al. constructed discrete [Mo_3_S_4_Fe] cubes, a molecular-level mimic of the FeMoco polyhedral core (Figure 3e) [48]. These synthetic metal–sulfur clusters feature Fe sites embedded within sulfur-rich Mo-based polyhedra, capable of capturing and reducing N_2_ via silylation. Under mild reductive conditions (Na/Me_3_SiCl), the system catalyzed N_2_ conversion to N(SiMe_3_)_3_, demonstrating true molecular-level N_2_ activation by synthetic Fe–S clusters. This work provides unprecedented evidence that Fe in sulfur-rich synthetic cubes can perform key steps in nitrogenase-like catalysis, validating the relevance of FeMoco-inspired geometry in small-molecule chemistry.

#### 4.1.4. Design Insights and Structure–Function Correlation

Collectively, these biomimetic studies highlight several converging design principles: (i) Fe/Mo bimetallic centers replicate the redox and orbital cooperativity in FeMoco; (ii) S-rich coordination frameworks emulate the cysteine-anchored environment of enzymes; (iii) atomic-scale dispersion and facet tuning allow for charge delocalization and intermediate stabilization; and (iv) cluster-like 3D architecture introduces spatial control over multi-site electron transfer [49,50]. These strategies open up new avenues for rational design of enzyme-like photocatalysts, with enhanced N_2_ adsorption, reduced activation energy, and favorable multi-electron pathways under solar illumination [51,52,53,54].

### 4.2. Mimicking Electron Transfer Pathways of Nitrogenase

In nitrogenase catalysis, electrons are shuttled from reduced ferredoxin or flavodoxin to the Fe protein, passed through the P-cluster (Fe_8_S_7_), and finally delivered to the active FeMoco site (MoFe_7_S_9_C). This spatially organized, redox-gated, and multistage electron transfer chain is central to the enzyme’s ability to overcome the thermodynamic barrier of N≡N triple bond cleavage. Mimicking this hierarchical electron delivery pathway—without the use of ATP—is a major goal for constructing efficient artificial photocatalysts.

#### 4.2.1. Single-Site Heterojunctions for Stepwise Electron Relay

**Figure 4:**
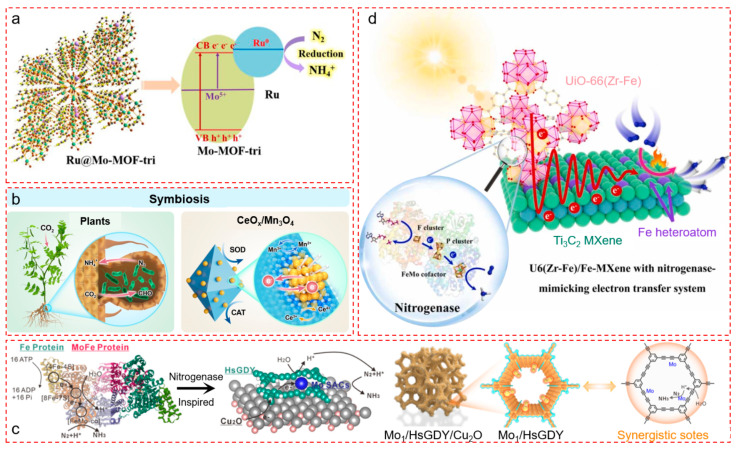
Bio-inspired design strategies for photocatalytic nitrogen fixation materials based on nitrogenase mimics. (**a**) Construction of single-site heterojunctions via Ru-anchored Mo-based MOFs enables precise energy band engineering. The coupling of Ru doping and Mo^5+^ defect states leads to enhanced light absorption and spatial charge separation, significantly boosting N_2_ activation. Reproduced with permission [55]. Copyright © 2022 American Chemical Society. (**b**) A nanozyme system mimicking plant–rhizobia symbiosis, constructed via confined CeO_x_ clusters anchored on Mn_3_O_4_ nanorods, achieves lattice interlacing and directional electron transfer (Mn^2+^→Ce^4+^), facilitating ROS scavenging and creating a protective redox microenvironment. Reproduced with permission [56]. Copyright © 2025 Wiley-VCH. (**c**) Schematic design of a nitrogenase-inspired photoelectrode based on Mo_1_/HsGDY@Cu_2_O, integrating defect-rich Mo sites with highly conjugated graphdiyne to form synergistic active centers for efficient solar-driven PEC-NRR. Reproduced with permission [57]. Copyright © 2025 American Chemical Society. (**d**) Rational assembly of UiO-66(Zr-Fe)/Fe-decorated MXene simulates the nitrogenase multi-site tandem pathway, with respective roles in photothermal conversion, electron transport, and N_2_ fixation. A high NH_3_ yield is achieved under solar irradiation in a gas–vapor–solid triphasic system. Reproduced with permission [58]. Copyright © 2024 Elsevier.

To reproduce such directional redox gradients, Wang et al. developed a metal–organic framework (MOF)-based photocatalyst by anchoring Ru single atoms onto a Mo-MOF with a [Mo_8_O_26_(im)_2_]^4−^ structure, forming Ruδ^+^–O_3_–Mo_3_ single-site heterojunctions (Figure 4a) [55]. These Ru–Mo ensembles mimic the P-cluster to FeMoco electron pathway by enabling sequential intraframework electron transfer. Energy band tuning was achieved by co-engineering Mo^5+^ defects and Ruδ^+^ active sites, enhancing visible-light absorption up to 700 nm and elevating the Fermi level, which reduces the energy barrier for *NNH formation—identified via DFT as the rate-limiting step. The system showed significantly higher activity than monodispersed analogs, highlighting the importance of site-specific redox heterojunctions in constructing enzyme-inspired relays.

#### 4.2.2. Electron-Confinement Nanozymes for Redox Cycling

While biological nitrogenase relies on precise protein–protein interfaces for electron transfer, Zhang et al. introduced a bio-inspired nanozyme architecture emulating this behavior through metal oxide–oxide coupling (Figure 4b) [56]. Their CeO_x_/Mn_3_O_4_ system mimics symbiotic electron transfer between rhizobia and plant cells. CeO_x_ nanoclusters (Ce^3+^/Ce^4+^ redox couple) are immobilized on root-like Mn_3_O_4_ supports (Mn^2+^/Mn^3+^ pair), enabling dynamic interfacial charge transfer analogous to enzyme–enzyme electron relay. The system exhibits catalase-like and superoxide dismutase-like activity, suggesting it can serve as a modular redox relay system in more complex N_2_ reduction cascades when adapted into multi-step photocatalytic platforms.

#### 4.2.3. Dual-Site Photocathodes for Synergistic H• and e^−^ Delivery

To simulate the proton/electron coupling mechanism of nitrogenase, Zhou et al. constructed a dual-site photocathode based on atomic Mo_1_ sites embedded in hydrogen-substituted graphdiyne (HsGDY) anchored onto Cu_2_O nanowires (Figure 4c) [57]. The HsGDY scaffold, with its extended π-conjugation and high proton conductivity, acts analogously to nitrogenase’s proton relay channels, enabling directed delivery of hydrogen radicals (H•). Meanwhile, the Mo_1_ centers offer preferential N_2_ adsorption and activation. Operando spectroscopy and DFT studies confirm that the Mo_1_–HsGDY interface provides a low-energy dual-pathway for N≡N hydrogenation. The device achieves a remarkable NH_3_ yield of 78.9 μg·cm^−^^2^·h^−^^1^ and Faradaic efficiency of 38.9% under 10-sun illumination—among the highest in photocathodic systems—showing that enzyme-inspired spatial cooperativity at the molecular level can be realized in scalable artificial systems.

#### 4.2.4. Hybrid Redox Scaffolds with Tandem Charge Flow

Extending this concept to hybrid materials, Tang et al. synthesized a composite catalyst comprising UiO-66(Zr–Fe) MOF integrated with Fe-dispersed MXene (Ti_3_C_2_) (Figure 4d) [58]. This architecture replicates the spatially resolved redox triad of nitrogenase: (i) UiO-66(Zr–Fe) provides photosensitization and initial electron generation; (ii) MXene nanosheets offer a conductive highway for charge migration; and (iii) atomically dispersed Fe sites act as terminal acceptors and N_2_ activators.

The resulting bi-phase gas–vapor–solid system achieves NH_3_ formation rates of up to 133.2 μmol·g^−1^·h^−1^ under full-spectrum light. The performance arises from effective photothermal coupling, multi-electron extraction, and minimized recombination loss, collectively emulating nitrogenase’s modular yet synchronized electron transfer system.

#### 4.2.5. Mechanistic Insights and Design Implications

Across these systems, several unifying design strategies emerge (Table 3).

These bio-inspired designs reinforce the importance of electron delivery geometry, energy level alignment, and dynamic valence control in constructing next-generation photocatalysts [59,60,61]. However, unlike nitrogenase, artificial systems lack real-time conformational flexibility, precise gating, and feedback control. Bridging these remaining gaps is the challenge addressed in the next section on energy-driven systems (Section 3.3) [62,63,64].

### 4.3. Artificial Energy Supply Systems: Toward ATP-Free Bio-Inspired Nitrogen Reduction

In the natural nitrogenase system, each electron transfer from Fe protein to the MoFe cofactor relies on the hydrolysis of two ATP molecules, which not only provides the energy required for bond rearrangement but also coordinates the spatiotemporal electron delivery essential for multi-step N_2_ fixation. Emulating this sophisticated biochemical regulation in artificial systems remains a fundamental challenge. Recent studies have explored ATP-free approaches by constructing multi-component light-driven platforms, quantum–biomolecule hybrids, and membrane-inspired architectures that collectively replicate the core functionalities of ATP-fueled electron transfer and energy modulation [16,17,19].

#### 4.3.1. Coupled Photocatalytic Hydrogen Generation and Nitrogen Reduction

**Figure 5:**
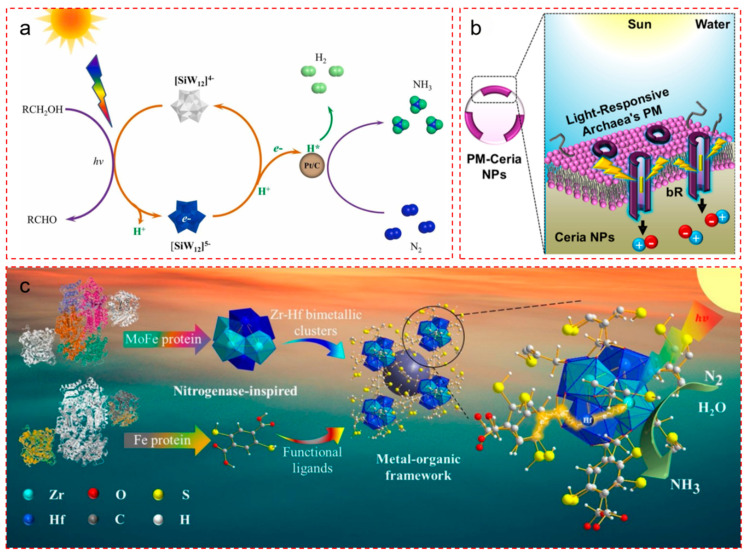
Biohybrid energy supply systems inspired by nitrogenase for light-driven nitrogen fixation under mild conditions. (**a**) A dual-catalyst photocatalytic system integrates polyoxometalate SiW_12_ as a photoredox mediator and Pt/C as the nitrogen hydrogenation site, enabling alcohol-driven in situ hydrogen generation and sustainable N_2_-to-NH_3_ conversion. Solar-driven dehydrogenation of alcohols supplies protons and electrons, which are stored in reduced SiW_12_ and subsequently transferred to Pt/C for N_2_ reduction via an associative distal pathway, bypassing the need for external H_2_. Reproduced with permission [65]. Copyright © 2024 Elsevier. (**b**) A photosynthetic biohybrid system (PBS) composed of ceria nanoparticles embedded within light-responsive purple membrane (PM) fragments from halobacterium salinarum. The bacteriorhodopsin chromophores (yellow rods) embedded in PM facilitate directional charge transfer under solar illumination, enabling efficient abiotic N_2_ reduction on ceria surfaces under ambient conditions. Reproduced with permission [66]. Copyright © 2025 American Chemical Society. (**c**) A nitrogenase-inspired metal-organic framework (MOF) catalyst, U(Zr-Hf)-2SH, integrates Zr-Hf bimetallic clusters with thiol-functionalized linkers. In this architecture, Zr sites serve as nitrogen activation centers while Hf acts as an electron reservoir, enabling a ligand-to-metal-to-metal electron transfer (LMMET) mechanism. The -SH-containing organic ligands extend visible-light absorption and promote π-backbonding with N_2_, collectively driving a high NH_3_ generation rate [67]. Copyright © 2021 Elsevier.

A promising approach involves dual-catalyst systems that spatially decouple light-induced electron generation and nitrogen hydrogenation. In a representative study by Zhang et al., a bio-inspired photocatalytic platform was developed using H_4_SiW_12_O_40_ (SiW_12_) as a redox-active photocatalyst and Pt/C nanoparticles as hydrogenation sites (Figure 5a) [65]. Here, renewable alcohols serve as the proton and electron source. Upon visible-light irradiation, SiW_12_ oxidizes alcohols and stores photogenerated electrons within its polyoxometalate anion structure. These stored electrons are subsequently transferred to Pt sites to reduce adsorbed N_2_ via an associative–distal mechanism.

Importantly, the system exhibits a markedly low energy barrier for N–H bond formation and is capable of producing [NNH_n_]^+^ and [NH_n_]^+^ intermediates under ambient conditions. By breaking the classical scaling relations through controlled redox mediation and electron flow separation, this approach simulates ATP-triggered FeMoCo electron influx with an artificial but robust electron reservoir.

#### 4.3.2. Semiconductor–Nitrogenase Biohybrids: Replacing ATP with Light-Driven Electron Transfer

A second approach directly couples photosensitizers with nitrogenase enzymes, offering a light-driven replacement for ATP hydrolysis. In a landmark work by Brown et al., CdS nanocrystals were used to photosensitize the MoFe protein of nitrogenase, forming a hybrid complex capable of catalyzing N_2_ reduction under illumination (Figure 5c,d) [68]. Here, light replaces ATP as the energy source, initiating site-specific electron transfer from CdS to MoFe clusters. The system achieved a turnover rate of 75 min^−1^, which is approximately 63% of that in ATP-coupled enzymatic conditions.

This hybrid model reproduces key features of the natural enzyme, including inhibition by CO and acetylene, confirming that the same mechanistic pathway is engaged. The work represents one of the most faithful mimics of biological nitrogen fixation using non-ATP pathways [67,69].

#### 4.3.3. Purple Membrane–Ceria Hybrids: Membrane-Inspired Electron Guidance

Beyond enzymatic strategies, Jang et al. reported a photosynthetic biohybrid system (PBS) that integrates free-standing ceria nanoparticles with the purple membrane (PM) of Halobacterium salinarum (Figure 5b) [66]. Even after separation from living cells, the PM retained the ability to direct photogenerated charge carriers unidirectionally. Electrostatic self-assembly between ceria and PM resulted in close interfacial contact, facilitating efficient N_2_-to-NH_3_ conversion under ambient pressure and temperature without requiring any toxic or precious metals.

In this construct, the Ce^3+^/Ce^4+^ redox pair serves as an intrinsic electron shuttle, while PM functions as a photonic membrane to regulate electron orientation. This platform mimics the spatial energy regulation provided by ATP binding and release cycles, enabling selective and efficient abiotic NRR in aqueous environments.

#### 4.3.4. Design Outlook

These three systems highlight complementary ATP-free routes toward biomimetic NRR catalysis. Key insights include the following: (i) Electron storage–release systems (e.g., polyoxometalates) can serve as artificial equivalents of ATP hydrolysis cycles. (ii) Semiconductor–enzyme hybrids allow light to directly fuel biological catalysts with preserved selectivity. (iii) Biohybrid membranes can emulate directional energy transfer and coupling through internal redox pairs [70,71].

Collectively, they embody a shift from energy-intensive thermal systems to self-sustained, ambient-condition NRR architectures that reflect the elegance of biological catalysis while advancing toward scalable application [72,73,74,75].

## 5. Interface Engineering and Defect Control in Biomimetic Photocatalysts

### 5.1. Bio-Inspired Structural Motifs: Bridging Atomic Architectures and Photocatalytic Activity

Nature’s nitrogen fixation mechanism, primarily driven by the FeMo cofactor in nitrogenase, has provided powerful inspiration for the design of heterogeneous photocatalysts [76]. This section summarizes recent progress in mimicking structural features of biological systems—such as coordination environments, vacancy architectures, and multi-metal active centers—to enhance N_2_ activation and hydrogenation efficiency under mild conditions [42].

#### 5.1.1. Zr–O Clusters in MOFs: Mimicking MoFe Binding Domains

**Figure 6:**
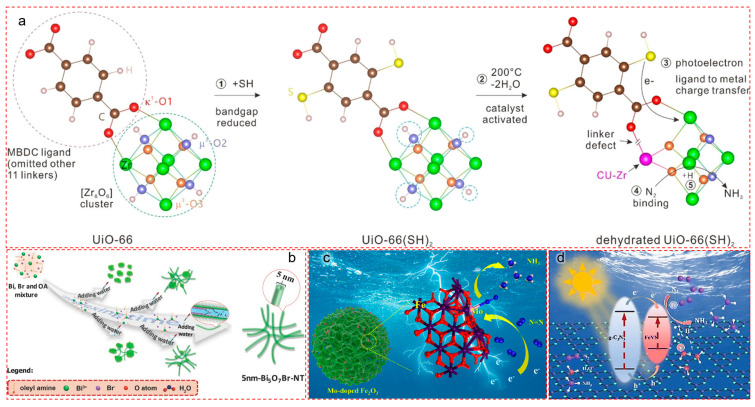
Bio-inspired defect engineering strategies enabling charge redistribution, electron localization, and N≡N bond activation in photocatalytic nitrogen fixation systems. (**a**) Stepwise structural evolution of UiO-66, UiO-66(SH)_2_, and its dehydrated form illustrating defect formation and photo-induced charge transfer. SH-functionalization lowers the bandgap, while dehydration triggers linker loss and generates Zr–coordinatively unsaturated sites (CUSs). These CUSs act as artificial enzyme-like coordination nodes, enabling μ-N–Zr interactions for visible-light-driven N_2_ reduction. Reproduced with permission [77]. Copyright © 2022 Wiley-VCH. (**b**) Schematic synthesis of ultrafine Bi_5_O_7_Br nanotubes from Bi–Br–oleylamine precursors. The tubular architecture enhances light absorption, exposes more defect-rich surfaces, and facilitates directional electron migration. Oxygen vacancies (OVs) at the tube wall act as N_2_ adsorption centers, simulating active FeMoco cavities. Reproduced with permission [78]. Copyright © 2017 Wiley-VCH. (**c**) Bio-inspired Mo-doped Fe_2_O_3_ porous nanospheres for electrochemical N_2_ reduction. Mo atoms replace Fe sites, forming Fe–O–Mo subunits resembling nitrogenase cofactors. The doping induces a porous morphology, defect generation, and favorable orbital redistribution. DFT simulations confirm N≡N bond elongation and a reduced barrier for the associative distal pathway. Reproduced with permission [79]. Copyright © 2022 American Chemical Society. (**d**) Proposed charge-separation mechanism in FeVS/C_3_N_4_ hybrid under visible light. The interface promotes spatially separated photogenerated electrons and holes, with electrons migrating toward FeVS to activate adsorbed N_2_. Conduction band alignment and defect-assisted transfer mimic the directional tunneling in enzymatic NRR. Reproduced with permission [80]. Copyright © 2023 Royal Society of Chemistry.

Guo et al. designed a UiO-66(SH)_2_-based MOF whose Zr_6_O_6_ cluster plays a role analogous to the MoFe cluster in nitrogenase, with a controlled dehydration step enabling μ-N–Zr interactions (Figure 6a) [77]. The stepwise weakening of the N≡N bond was attributed to photo-induced electrons facilitating successive protonation inside the [Zr_6_O_6_] cage. In situ EXAFS and TGA confirmed structural dynamics post-dehydration, and ^15^N_2_ isotopic labeling validated ammonia as the exclusive product. This study highlights how metal–oxygen polyhedra can be designed to mimic the reaction cavity and binding specificity of metalloenzymes.

#### 5.1.2. Light-Switchable Oxygen Vacancies in Bismuth-Based Nanotubes

Inspired by enzyme flexibility and redox plasticity, Wang et al. created ultrathin Bi_5_O_7_Br nanotubes (5 nm) with controllable surface oxygen vacancies (OVs), which act as dynamic electron reservoirs for activating N_2_ (Figure 6b) [78]. These light-switchable OVs enabled a high ammonia yield of 1.38 mmol h^−1^ g^−1^ and a quantum efficiency exceeding 2.3% under 420 nm illumination in pure water. The unique tubular morphology and large surface area promote N_2_ adsorption, while visible-light-induced OVs simulate the redox plasticity of nitrogenase. This work offers an elegant example of mimicking the electron-rich environments of enzyme pockets using nanostructured defect sites.

#### 5.1.3. Mo-Doped Hematite: Electrochemical Analog of FeMo Cofactor

Niu et al. synthesized Mo-doped α-Fe_2_O_3_ porous nanospheres exhibiting Fe–O–Mo subunits that emulate the electronic synergy of the FeMo cofactor (Figure 6c) [79]. Mo doping modified the morphology and enriched defect density, enhancing N_2_ chemisorption and electron conductivity. Electrochemical NRR experiments revealed a Faradaic efficiency of 11.2% and NH_3_ yield of 21.3 ± 1.1 μg h^−1^ mg_cat_^−1^ under ambient conditions. DFT simulations showed that Mo doping elongated the N≡N bond (1.132 Å) and favored an associative distal pathway. This result suggests that atomic-level heteroatom engineering is essential to translate enzymatic motifs into scalable electrocatalytic platforms [18,33].

#### 5.1.4. Polysulfide-Based Bimetallic Centers: Synergistic Fe–V Activation

Cai et al. constructed a biomimetic photocatalyst by incorporating a vanadium-doped iron polysulfide active center onto nitrogen-deficient g-C_3_N_5_, mimicking the multi-metal synergism of nitrogenase (Figure 6d) [80]. While Fe in the polysulfide component preferentially adsorbed dinitrogen, V facilitated water activation and proton transfer. This dual-function system showed significantly boosted N_2_ fixation activity (1916.5 μmol h^−1^ g^−1^), 6.81 times higher than the pristine g-C_3_N_5_. The spatial separation of N_2_ activation and protonation zones emulates enzymatic pathways and reduces charge recombination [81].

#### 5.1.5. Design Implications

These examples illustrate how defect engineering can serve biomimetic functions: (i) electronic tuning of the band edge via Ov/Nv/Sv; (ii) dual-site chemisorption for cooperative N_2_ binding and hydrogenation; (iii) formation of enzyme-like cavities that regulate intermediate stabilization; and (iv) charge localization and multi-electron storage, mimicking enzymatic redox buffering [82,83,84].

As shown in Figure 4, the above defect-engineered materials introduce localized asymmetry, orbital perturbation, and catalytic specificity that resemble nitrogenase cofactor microenvironments. In Section 4.2, we explore how interfacial regulation further enhances these effects by creating heterojunctions and cooperative interfaces [85,86].

### 5.2. Interfacial Engineering: Constructing Functional Junctions for Catalytic Enhancement

In biological nitrogen fixation, interprotein interfaces play an indispensable role in ensuring spatiotemporal control of electron delivery and protonation. Inspired by this [16,17,19], artificial photocatalytic systems have focused on constructing heterointerfaces to manipulate charge separation, regulate reaction energetics, and enable dual-function catalysis [44].

#### 5.2.1. Biological Membrane–Nanoparticle Hybrid: Interface-Guided Redox Modulation

**Figure 7:**
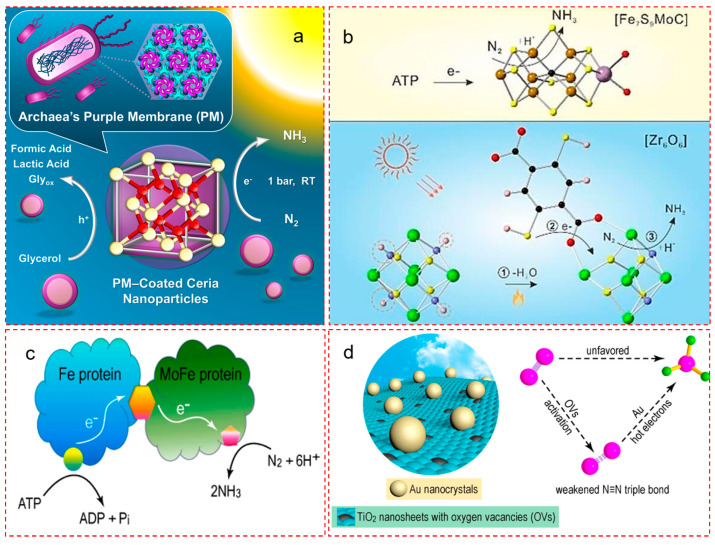
Biomimetic interfacial engineering strategies for enhanced photocatalytic nitrogen fixation. (**a**) Schematic of a purple-membrane (PM)-coated CeO_2_ nanoparticle system mimicking light-driven ATP-free proton/electron coupling for N_2_ reduction. The archaeal PM acts as a bio-light harvester, enabling photogenerated electrons to reduce N_2_ on ceria surfaces via an enzyme-like multi-channel reaction pathway. Organic acids (formic acid, lactic acid, glycerol) serve as hole scavengers to maintain redox balance. Reproduced with permission [66]. Copyright © 2025 American Chemical Society. (**b**) Comparison of enzymatic and artificial coordination environments. The upper panel illustrates the Fe–S–Mo–C cofactor in nitrogenase with ATP-powered electron transfer for *N_2_ reduction, while the lower panel shows a dehydrated Zr_6_O_6_-cluster-based MOF (UiO-66-(SH)_2_) with μ-N–Zr binding, enabling visible-light-driven NRR via a proton-assisted multi-electron process. Reproduced with permission [81]. Copyright © 2025 Wiley-VCH. (**c**) Schematic of the natural nitrogenase complex comprising the Fe protein and MoFe protein. ATP hydrolysis induces conformational changes at the docking interface, enabling stepwise electron delivery from the Fe protein to the MoFe protein and finally to *N_2_ for reduction to NH_3_. Reproduced with permission [88]. Copyright © 2018 American Chemical Society. (**d**) A tandem-interface photocatalyst composed of Au nanocrystals anchored on ultrathin TiO_2_ nanosheets with abundant oxygen vacancies (OVs). Hot electrons from surface plasmon resonance (Au) and chemisorption activation at OVs synergistically weaken the N≡N bond, facilitating selective NH_3_ formation. Apparent quantum efficiency reaches 0.82% at 550 nm, highlighting the catalytic advantages of interface synergy and visible-light harvesting. Reproduced with permission [88]. 2018 American Chemical Society.

To extend interfacial engineering into hybrid bioinorganic systems, Jang et al. developed a photosynthetic biohybrid system (PBS) by integrating free-standing CeO_2_ nanoparticles into purple membranes (PMs) derived from Halobacterium salinarum (Figure 7a) [66]. Unlike the previous structural focus in Section 3.3, the emphasis here is on interface-directed charge modulation [30,87].

The electrostatic interaction between PM and CeO_2_ established unidirectional electron transfer channels under visible light, enabling efficient photoreduction of N_2_ to NH_3_ at room temperature and atmospheric pressure—without requiring noble metals or enzyme reconstitution. This interfacial assembly mirrors the protein–cofactor coupling in natural nitrogenase, offering a stable and tunable microenvironment that enables redox versatility while preserving catalytic integrity [18,33].

#### 5.2.2. In-Lattice Heterojunctions: Atomic-Level Carrier Highways

Chen et al. reported an “in-lattice heterojunction” photocatalyst by embedding Fe–N_4_-anchored carbon (Fe–N–C) layers into the cavity of defective TiO_2_ (D-TiO_2_), forming a confined Fe–N–C@D-TiO_2_ structure with atomic continuity along the Ti–C–N–Fe axis (Figure 7b) [81]. This arrangement established radial charge carrier migration pathways with minimized recombination and enhanced electronic communication [25].

More importantly, the tailored heterojunction promoted N_2_ adsorption and activation at Fe–N_4_ centers, leading to an ammonia yield of 88 μmol g^−1^ h^−1^—significantly surpassing that of non-cavity Fe–N–C catalysts. The lattice-confined configuration mimics the biological Fe protein–MoFe protein complex in terms of confined spatial orientation and charge steering via coordinated atomic channels [25,30].

#### 5.2.3. Tandem Interfaces: Hot Electron-Driven Multistage Catalysis

Yang et al. introduced a tandem photocatalytic system consisting of plasmonic gold nanocrystals (Au NPs) anchored on ultrathin TiO_2_ nanosheets with abundant oxygen vacancies (OVs) (Figure 7c,d) [88]. The system exhibited dual interfacial functionality: the TiO_2_ vacancies chemisorbed and activated N_2_ molecules, while hot electrons generated by localized surface plasmon resonance (LSPR) of Au NPs reduced the activated intermediates [58].

This “working-in-tandem” configuration led to a high ammonia production rate with an apparent quantum efficiency of 0.82% at 550 nm. Moreover, optimized combinations of Au nanospheres and nanorods enhanced broadband light utilization, improving the N_2_ fixation rate by 66.2%. The spatial division of excitation and activation resembles enzymatic compartmentalization within nitrogenase’s multi-domain architecture [29,69].

#### 5.2.4. Facet-Dependent Electronic Structures and N_2_ Adsorption

Different crystal facets of a photocatalyst expose specific atomic terminations, which define surface energy, electron density, and adsorbate binding behavior [29]. Li et al. constructed {001}/{010} co-exposed BiVO_4_ nanosheets and found that the {010} facet promotes charge accumulation, while the {001} facet preferentially adsorbs N_2_, leading to spatially resolved *N_2_ reduction and *H_2_O oxidation [44]. This mirrors how FeMoco’s geometry creates selective substrate tunnels to separate proton and N_2_ channels [89,90]. Similarly, Liu et al. synthesized TiO_2_ nanocrystals with dominant {101} facets, which display enhanced *N_2_ chemisorption and lower work function compared to {001} facets [83]. This reflects how enzymes use facet-like pocket polarity to achieve differential substrate interactions [91].

#### 5.2.5. Summary of Biomimetic Interfacial Strategies

Across these representative systems, several biomimetic features emerge: (i) atomic-scale charge migration pathways, emulating protein electron tunnels; (ii) dual-function tandem interfaces, enabling sequential excitation and activation; and (iii) biohybrid membranes, integrating organic–inorganic interfaces for redox control [29,69]. Together, these strategies reflect the essence of biological nitrogen fixation interfaces—spatial precision, redox flexibility, and selective electron–proton coupling—offering insights for future design of efficient N_2_ fixation catalysts under ambient conditions [92,93].

## 6. Conclusions and Outlook

Biomimetic photocatalytic nitrogen fixation represents a compelling strategy to bridge the performance chasm between natural enzymatic nitrogenase systems and artificial N_2_-to-NH_3_ conversion technologies. Through rational mimicry of structural, electronic, and functional features of nitrogenase, recent advances have demonstrated how catalyst design can transcend traditional semiconductor limitations and approach biological efficiency and selectivity under ambient conditions [94].

This review systematically dissects six core bio-inspired design strategies (Figure 2, Figure 3, Figure 4, Figure 5 and Figure 6): (i) active site mimicry (Fe–S, Mo–Fe–C, Fe-only cofactor models) reconstructs catalytic cores akin to FeMoco [43,44,45,65,77]; (ii) electron transfer pathways simulate P-cluster → FeMoco relay cascades via π-conductive networks and redox modules [55,58,59,60]; (iii) artificial energy supply systems bypass ATP via plasmonic, redox cycling, and light-driven donor–acceptor modules [60,65,66,68]; (iv) defect engineering introduces asymmetry, charge localization, and dual-site activation resembling enzymatic pocket dynamics [76,77,79,80,81]; (v) interfacial regulation mimics enzymatic docking, redox cooperativity, and molecular orientation via semiconducting–molecular hybrids [44,58,78,88]; and (vi) facet and spatial confinement strategies replicate enzymatic channel gating, microenvironmental steering, and intermediate stabilization [44,48,66,81].

Despite these achievements, several fundamental challenges remain: (i) Inadequate dynamic modulation: Unlike nitrogenase, which orchestrates redox steps via ATP-induced conformational gating [18,20,33], most artificial systems lack temporal regulation mechanisms. Static structures fail to match the dynamic demands of proton–electron coupling required for multi-step N_2_ reduction [25,69]. (ii) Ambiguity in reaction mechanism: The exact activation route—associative distal, alternating, or enzymatic hybrid pathways—often remains speculative. Although DFT modeling is widely used [60,79,81], experimental evidence such as isotope tracing ([^15^N], [^1^H] NMR), in-situ XAFS/FTIR, and operando spectroscopy is still scarce [26,69,87]. (iii) Poor integration in system-level devices: Most reported photocatalytic systems operate in suspension or half-cell modes. Translation into scalable, stable device platforms (e.g., flow reactors, PEC systems) is minimal. Integration with solar-to-ammonia systems or bio-hybrid platforms (quantum dot–enzyme conjugates [68]) remains in early stages.

To push the frontier of biomimetic photocatalytic NRR, future research may focus on the following: (i) Dynamic Interface Engineering: Develop stimuli-responligands, pH-sensitive redox gates) that mimic ATP-regulated Fe-protein docking [17,19]. Temporal control of electron flux may drastically improve N_2_RR turnover efficiency. (ii) Mechanistic Decoding by Multimodal Characterization: Combine DFT with operando Raman, Mössbauer, and isotopic tracing to distinguish real intermediates (e.g., *NNH, *NH_2_) and validate simulated reaction coordinates. Synchronizing theory and spectroscopy will clarify pathway ambiguity [26,29,87]. (iii) Biohybrid and Device Integration: Construct MOF–nanozyme–semiconductor hybrids or quantum dot–cofactor mimetics that maintain enzyme-like function within scalable architectures. Integrate into photochemical or PEC systems with bias-free N_2_RR, approaching real-world application. (vi) AI-Guided Catalyst Design: Utilize machine learning with descriptors such as Bader charge, orbital occupancy, and vacancy formation energy to guide cofactor-level screening and facet–defect–interface synergies.

In summary, although each strategy provides unique advantages, hybrid approaches that combine the structural fidelity of biomimetic catalysts, the scalability of semiconductor vacancy engineering, and the directional charge transfer of biohybrids appear most promising. Particularly, atomic-level site engineering and tandem plasmonic–semiconductor systems stand out for bridging efficiency and scalability. The convergence of these approaches with advanced in situ characterization, AI-driven screening, and device-level integration may yield the next generation of nitrogenase-inspired catalysts capable of sustainable ammonia synthesis under ambient conditions.

## Figures and Tables

**Table 1:** Comparison of Haber–Bosch process and bio-inspired photocatalytic nitrogen fixation in terms of yield and cost.

Parameter	Haber–Bosch	Bio-Inspired Photocatalysis
Typical operating conditions	400–500 °C, 150–300 bar	Ambient temperature and pressure
Yield scale	1500–2000 tons NH_3_ per day	μmol–mmol NH_3_ g^−1^ h^−1^
Energy cost	8–12 MWh per ton NH_3_	Mainly solar input
CO_2_ emissions	1.6–2.0 tons CO_2_ per ton NH_3_	Near-zero (if powered by renewables)
Cost per ton (USD)	300–600 (fossil-based H_2_)	Currently not scalable; projected competitive if ≥1–2% solar-to-ammonia efficiency is reached

**Table 2:** Benchmark comparison of N_2_-to-NH_3_ systems.

System	Rate/Efficiency	Conditions	Advantages	Limitations
**Nitrogenase (MoFe protein)**	~10–20 NH_3_ s^−1^ per enzyme (~10^−2^ mmol g^−1^ h^−1^); Faradaic efficiency ~ 60–70%	Ambient T, P; ATP-driven	Operates under mild conditions, high selectivity	Requires 16 ATP per N_2_, slow compared to industrial process
**Haber–Bosch**	>1000 mmol g_cat_^−1^ h^−1^ (industrial scale); ~10–15% energy efficiency	400–500 °C, 150–300 atm, Fe/Co catalysts	Scalable, mature, >150 Mt NH_3_ annually	Enormous energy input, CO_2_ emissions
**Artificial photocatalysts (recent)**	10–500 μmol g^−1^ h^−1^; quantum efficiency up to 2%	Ambient T, P, solar-driven	Mild, renewable-driven, tunable selectivity	3–5 orders slower than Haber–Bosch; stability challenges

**Table 3:** Comparative mapping of key design elements in biological nitrogenase versus artificial biomimetic photocatalysts.

Design Element	Biological Counterpart	Artificial Mimic
Stepwise redox sites	P-cluster → FeMoco	Ruδ^+^–Mo^5+^ pairs in MOFs [55]
Redox cycling via metal valence	Protein-based switching	Ce^3+^/Ce^4+^–Mn^2+^/Mn^3+^ relay [56]
Directional H•/e^−^ supply	Proton-coupled electron relay	HsGDY–Mo_1_ dual site [57]
Tandem charge transport	Ferredoxin to FeMoco	UiO-66/Fe–MXene relay [58]

## Data Availability

Not applicable.
